# Exploring the mechanism of Danggui Buxue Decoction in regulating atherosclerotic disease network based on integrated pharmacological methods

**DOI:** 10.1042/BSR20211429

**Published:** 2021-10-12

**Authors:** Hao Xu, Tianqing Zhang, Ling He, Mengxia Yuan, Xiao Yuan, Shanshan Wang

**Affiliations:** 1 School of Integrated traditional Chinese and Western Medicine, Hunan University of Chinese Medicine, Changsha, Hunan Province, China; 2 Department of Cardiology, The First Affiliated Hospital of University of South China, Hengyang, Hunan Province, China; 3 Department of Infectious Diseases, The First Affiliated Hospital of University of South China, Hengyang, Hunan Province, China; 4 Shantou University Medical College, Shantou University, Shantou, Guangdong Province, China

**Keywords:** Atherosclerosis, Bioinformatics, Chinese Medicine, Danggui Buxue Decoction, Herb Medicine, Integrated pharmacological

## Abstract

**Objective:** To explore the mechanism of Danggui Buxue Decoction (DGBXD) in regulating Atherosclerosis (AS) network based on integrated pharmacological methods.

**Methods:** The active ingredients and targets of DGBXD are obtained from TCMSP database and ETCM. AS-related targets were collected from the Genecards and OMIM databases. The drug–disease protein interaction (PPI) networks were constructed by Cytoscape. Meanwhile, it was used to screen out densely interacting regions, namely clusters. Finally, Gene Ontology (GO) annotations are performed on the targets and genes in the cluster to obtain biological processes, and Kyoto Encyclopedia of Genes and Genomes (KEGG) annotations are performed on the targets of the PPI network to obtain signaling pathways.

**Results:** A total of 212 known targets, 265 potential targets and 229 AS genes were obtained. The ‘DGBXD known-AS PPI network’ and ‘DGBXD-AS PPI Network’ were constructed and analyzed. DGBXD can regulate inflammation, platelet activation, endothelial cell apoptosis, oxidative stress, lipid metabolism, vascular smooth muscle proliferation, angiogenesis, TNF, HIF-1, FoxO signaling pathway, etc. The experimental data showed that compared with the model group, the expressions of ICAM-1, VCAM-1, and interleukin (IL)-1β protein and mRNA in the DGBXD group decreased (*P*<0.05). However, plasma IL-1β, TNF-α, and MCP-1 in the DGBXD group were not significantly different from the model group (*P*>0.05).

**Conclusion:** The mechanism of DGBXD in the treatment of AS may be related to the improvement of extracellular matrix (ECM) deposition in the blood vessel wall and the anti-vascular local inflammatory response, which may provide a reference for the study of the mechanism of DGBXD.

## Introduction

Atherosclerosis (AS) is a common disease that seriously harms human health, and is the most common and important type of arteriosclerosis [1,2]. There are lipid deposits in the arterial intima, accompanied by the proliferation of smooth muscle cells (SMCs) and connective tissue, which results in the formation of fibrous plaques on the intima, causing the vessel wall to thicken, harden, and narrow the lumen. Then, the connective tissue that deposits a large amount of lipids in the plaque undergoes necrosis to form atheroma [2,3]. AS mainly involves large- and medium-sized arteries, namely the aorta and its main branches (brain, kidney, arteries of the limbs, coronary arteries, etc.) [3]. The etiology and pathogenesis of AS have not yet been fully understood, but it is unanimously recognized that hyperlipidemia, smoking, and hypertension are the main risk factors for the disease [4,5].

The current treatment measures are mainly lipid-lowering, anticoagulant and thrombolytic drugs, but the treatment effect is not ideal due to poor compliance and drug side effects [6,7]. Danggui Buxue Decoction (DGBXD) is composed of *Angelicae Sinensis Radix* (Danggui) and *Hedysarum Multijugum Maxim.* (Huangqi) [8]. Modern pharmacological research shows that DGBXD has pharmacological effects such as improving blood rheology and hemodynamics, regulating blood lipids, improving vascular endothelial function, and regulating inflammation. It has a significant effect on the treatment of hyperlipidemia, coronary heart disease, cardiac neurosis, bi-heart disease, and brain–heart syndrome [9–12]. In recent years, clinical and experimental studies have shown that DGBXD can improve AS by inhibiting inflammation, protecting endothelial cell function, and improving hemodynamics [9–12]. However, the therapeutic effect and mechanism of DGBXD on AS are still unclear. In view of the multi-component, multi-effect, multi-target and overall regulatory effects of traditional Chinese medicine (TCM), the present study uses TCM to integrate pharmacological strategies to explore the key targets and signal pathways of DGBXD intervention in AS, to explore its molecular mechanism, in order to provide a basis for the development and development of DGBXD drugs [13].

**Figure 1:**
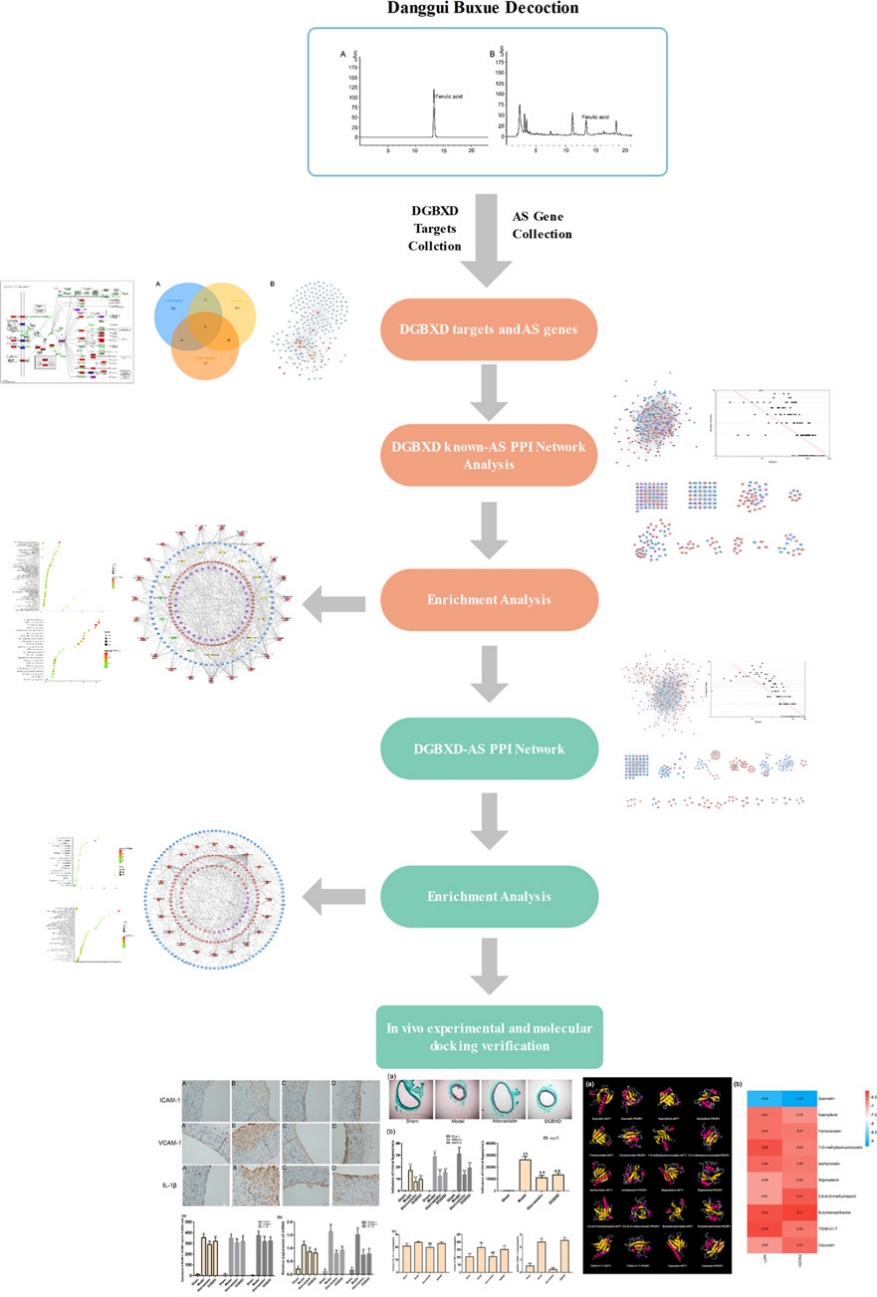
The idea and process of this research

Integrated pharmacology is a new model of modern TCM research [14]. The law of interaction between the substance entity of TCM prescriptions and the body is one of the key scientific issues in the study of integrated pharmacology. It is an interdisciplinary integration of TCM chemistry, pharmacokinetics, pharmacology, systems biology, and computational science [15]. Our previous research has applied integrated pharmacology to the herbal formulae to intervene in cardiovascular, tumor, and endocrine diseases by developing new methodology [16–18]. Therefore, this study will explore the mechanism of DGBXD on AS through integrated pharmacological strategies. The idea and process of this research was shown in Figure 1.

## Materials and methods

### Known and potential targets collection

The components and known targets of DGBXD were collected from TCMSP database (http://tcmspw.com/index.php) [19] with their oral bioavailability (OB) ≥ 30%, Caco-2 permeability > −0.4 and drug-likeness (DL) ≥ 0.18 (Table 1). The potential targets were collected from ETCM (http://www.tcmip.cn/ETCM/index.php) [20], which is a database including comprehensive and standardized information for the commonly used herbs and formulas of TCM, as well as their ingredients (Supplementary Tables S1 and S2).

**Table 1:** The potential components

Molecule name	MW	OB (%)	Caco-2	DL
(3R)-3-(2-hydroxy-3,4-dimethoxyphenyl)chroman-7-ol (64474-51-7)	302.35	67.67	0.96	0.26
(3S,8S,9S,10R,13R,14S,17R)-10,13-dimethyl-17-[(2R,5S)-5-propan-2-yloctan-2-yl]- 2,3,4,7,8,9,11,12,14,15,16,17-dodecahydro-1H-cyclopenta[a]phenanthren-3-ol (64997-52-0)	428.82	36.23	1.45	0.78
(6aR,11aR)-9,10-dimethoxy-6a,11a-dihydro-6H-benzofurano[3,2-c]chromen-3-ol (73340-41-7)	300.33	64.26	0.93	0.42
1,7-Dihydroxy-3,9-dimethoxy pterocarpene	314.31	39.05	0.89	0.48
3,9-di-*o*-methylnissolin	314.36	53.74	1.18	0.48
7-*o*-methylisomucronulatol	316.38	74.69	1.08	0.3
β-sitosterol	414.79	36.91	1.32	0.75
Bifendate	418.38	31.1	0.15	0.67
Calycosin	284.28	47.75	0.52	0.24
Formononetin	268.28	69.67	0.78	0.21
Hederagenin	414.79	36.91	1.32	0.75
Isoflavanone	316.33	109.99	0.53	0.3
Isorhamnetin	316.28	49.6	0.31	0.31
Jaranol	314.31	50.83	0.61	0.29
Kaempferol	286.25	41.88	0.26	0.24
Mairin	456.78	55.38	0.73	0.78
Quercetin	302.25	46.43	0.05	0.28
Stigmasterol	412.77	43.83	1.44	0.76
Ferulic acid	194.2	39.56	0.47	0.06
Butylidenephthalide	188.24	42.44	1.32	0.07
Senkyunolide I	204.24	46.8	0.87	0.08

### AS gene collection

The AS-related genes were collected from Genecards (http://www.genecards.org) [21] and Online Mendelian Inheritance in Man (OMIM) (http://omim.org/) databases [22] with keyword ‘Atherosclerosis’. The genes with relevance score ≥ 6.0 were selected for sequence research (Supplementary Table S3).

### Network construction and analysis methods

The protein–protein interaction (PPI) data of DGBXD targets and AS genes were collected from String 11.0 (https://string-db.org) [23]. According to DGBXD target and AS gene information, Cytoscape 3.7.0 software is used to construct a drug target–disease gene network (i.e. DGBXD Known Target-AS PPI network and DGBXD-AS PPI network) [24]. Then, the DGBXD–AS PPI network was analyzed by the ‘Network Analyzer’ and ‘MCODE’ to collect the degree and betweenness of nodes and the cluster of this PPI network [24]. The DAVID ver 6.8 (https://david.ncifcrf.gov/) was utilized to perform Kyoto Encyclopedia of Genes and Genomes (KEGG) pathway enrichment analysis and Gene Ontology (GO) enrichment analysis [25].

### Molecular docking analysis

The molecular structure of DGBXD components were collected from TCMSP. The PDB database (https://www.rcsb.org/) was used to retrieve the 3D structure of PIK3R1 (PDB ID: 1H9O) and AKT1 (PDB ID: 1H10), and downloaded the file in the ‘pdb’ format [26]. Discovery Studio Client Ver. 4.5 was used to hydrogenate, remove water, and remove ligand molecules from receptor molecules. Auto Dock ver. 4.2 software was used for molecular docking, supplemented by SwissDock [27]. If the binding energy of the receptor and the ligand is ≤ −5.0 kcal/mol, it is considered that the ligand can bind to the receptor stably [28,29].

### Experimental materials

#### Experimental animals

Forty (40) specific pathogen-free (SPF) Sprague–Dawley (SD) male rats, weighing 220–250 g, were purchased from Hunan Slack Jingda Experimental Animal Co., Ltd. [Quality Certificate Number: SCXK (Xiang) 2013-0004]. The rats were bred adaptively for 1 week before the experiment with a humidity of 45–65% and a room temperature of 25°C. All animal experiments took place at the Experimental Animal Center of Hunan University of Chinese Medicine, License number: SCXK (Xiang) 2013-0005. Animal experiments were approved by the Animal Ethics Committee of Hunan University of Chinese Medicine (Ethical approval number: HUCM-15021) and were in accordance with the National Institute of Health’s Guide for the Care and Use of Laboratory Animals.

#### Experimental drugs

*Angelicae Sinensis Radix* is produced in Gansu Province (batch number: 20170501); *Hedysarum Multijugum Maxim*. is produced in Neimenggu Province (batch number: 20171607). Herbs were purchased by the Department of Pharmacy of the First Affiliated Hospital of Hunan University of Chinese Medicine and appraised by Professor Zuo Yajie of the Department of Pharmacy of the First Affiliated Hospital of Hunan University of Chinese Medicine. Atorvastatin calcium is produced by Zhejiang Xindonggang Pharmaceutical Co., Ltd. (batch number: 20160803, specification: 10 mg/tablet).

#### Reagents and instruments

Mouse anti-rat intercellular cell adhesion molecule-1 (ICAM-1) monoclonal antibody, rabbit anti-rat vascular cell adhesion molecule-1 (VCAM-1) monoclonal antibody, rabbit anti-rat interleukin-1β (IL-1β) polyclonal antibodies were purchased from Abcam company. DAB color reagent kit (batch number: SP-900D) and immunohistochemical staining kit (batch number: SP-9001) were purchased from Beijing Zhongshan Jinqiao Biological Co., Ltd. Rat tumor necrosis factor-α (TNF-α) ELISA kit (lot number: E-EL-R0019c), rat monocyte chemotactic protein-1 (MCP-1) ELISA kit (lot number: E-ELR0633c), rat IL-1β ELISA kit (batch number: E-EL-R0012c) were purchased from Elite Biotech Co., Ltd. Ferulic acid reference substance (batch number 0773-9910, for content determination) was purchased from China Institute for the Control of Pharmaceutical and Biological Products.

The 2.0 mm × 15 mm Runjin medical balloon catheter, Runthrough guide wire were purchased from Japan Terumo Co., Ltd., and the medical balloon dilatation pressure pump was purchased from the Department of Apparatus, Jiangxi Provincial Hospital of Traditional Chinese Medicine. A 1260 type High Performance Liquid Chromatograph y(HPLC) (Aglient Company), Waters 2996 Diode Array Detector (PDA); Model 98-1-B Heating Mantle (Tianjin Test Instrument Co., Ltd.); Model N-1000 Rotary Evaporator (RIKAKIKAI Company)were used.

### Experimental methods

#### Preparation of drugs

The herbs were accurately weighed and extracted three times by hydrothermal reflux method (the first time was extracted with eight times the amount of water for 1 h; the second and third times were extracted with six times the amount of water for 1 h). The extracts of three times were combined and filtered. Then, the extracts was evaporated and concentrated under vacuum at 60°C to make the DGBXD extracts, and the DGBXD extracts were taken out to a constant volume with distilled water 0.39 g of crude drug/ml. A 0.1% sodium benzoate is added to the medicinal solution and stored in a refrigerator at −4°C for later use.

#### Animal modeling, grouping, and intervention

The current balloon injury model is a hyperplasia/neointima model, and it is also a mainstream model in the early stage of AS [30]. After the rats were anesthetized with 4% sodium pentobarbital (50 mg/kg), the left common carotid artery was surgically exposed. The distal end of the left common carotid artery was ligated, the proximal end was clamped by an arterial clip, and then a ‘V’-shaped incision was made at the distal end. The balloon tube was then inserted through the incision, crossing the aortic arch to a depth of approximately 6–7 cm. The balloon was pressurized with a medical balloon expansion pressure pump to maintain the pressure at 8 bar, and the balloon catheter was repeatedly pulled back and forth to the aortic arch four times. Then the balloon catheter was rotated to 180° and the same operation was performed four times. After the completion of the balloon expansion pressure pump back to the negative pressure state, the catheter was withdrawn.

The rats were randomly divided into four groups: sham operation group, model group, DGBXD group, and atorvastatin group. According to the pre-experimental modeling situation, each group contained eight to ten rats. Sham operation group: only the left common carotid artery was separated and exposed and then sutured without balloon injury; the same amount of distilled water was given after the operation. Model group: balloon injury of the thoracic and abdominal aorta was performed without drug intervention after operation; the same amount of distilled water was given after operation. Atorvastatin group: after thoracic and abdominal aortic balloon injury, atorvastatin was administered. DGBXD group: after thoracic and abdominal aortic balloon injury, the DGBXD 3.9 of crude drug g/kg was administered.

#### Specimen collection

After 14 days of intragastric administration (the day after the last administration), blood was collected from the abdominal aorta under anesthesia with 4% sodium pentobarbital (50 mg/kg), and then the thoracic and abdominal aortic vessels were intercepted. After that, the rats were sacrificed by cervical dislocation. After the blood was centrifuged at low temperature (4°C, 2000 rpm for 15 min), the plasma was collected and stored in a refrigerator at −80°C for ELISA testing. The blood vessels used for immunohistochemical detection were fixed with 4% paraformaldehyde and stored in a refrigerator at 4°C.

#### Vascular intimal hyperplasia index measurement

After the thoracic-abdominal aorta was taken out, it was rinsed with normal saline to remove the connective tissue outside the blood vessel, and fixed in 4% paraformaldehyde for 24 h. Then, the ethanol gradient dehydration was carried out, and the paraffin was embedded vertically, and eight slices of each segment of blood vessel were cut uniformly for Masson’s staining. After staining, observe under a light microscope, and the MIAS medical image analysis system was used to take pictures. Image-Pro Plus6.0 image analysis software is used to measure the media area (MA), the perimeter of the midline of the media, the intimal area (IA), and the perimeter of the midline of the intima. Media thickness (MT) = Media area/Median midline circumference. Intimal thickness (IT) = intimal area/intimal midline circumference. Hyperplasia ratio of intimal area (HRIA) and hyperplasia ratio of intimal thickness (HRIT) were also calculated.

#### Detection of ICAM-1, VCAM-1, IL-1β mRNA expression in the intima of hyperplastic vessels

The total RNA of the aortic tissues of each group was extracted by TRIzol method. Then reverse transcription of RNA into cDNA was performed according to the operating instructions of the kit. The RT-PCR was performed using the two-step chimeric fluorescence quantitative RT-PCR kit (TaKaRa Company, SYBR Green chimeric fluorescence method). RT-PCR conditions: reverse transcription reaction in a constant temperature water bath was performed at 37°C for 15 min; then it was placed in 85°C water for 5 s to inactivate the reverse transcriptase; Rotor-Gene 3000 Real-Time PCR amplification analysis system was used for RT-PCR. Reaction conditions (40 cycles in total): pre-denaturation 95°C for 30 s, denaturation 95°C for 10 s, annealing 60°C for 30 s, extension 72°C for 30 s, extension 72°C for 5 min. The primers were synthesized by Shanghai Bioengineering Company and passed quality inspection (Table 2). The relative expression of target gene mRNA was calculated by 2^−ΔΔ*C*_t_^.

**Table 2:** The primers

Gene	Sequence	Length/bp
*IL-1β*	F: 5′-CCTGTGGCCTTGGGCCTCAA-3′	204
	R: 5′-GGTGCTGATGTACCAGTTGGG-3′	
*ICAM-1*	F: 5′-GCGGCCTTGGAGGTGGAT-3′	485
	R: 5′-GGAGGCGGGGCTTGTACC-3′	
*VCAM-1*	F: 5′-CCTGTCCCAGAGGAGGGC-3′	500
	R: 5′-CAACTGCGAGCCGACTTCG-3′	
*β-actin*	F: 5′-AGCTGAGAGGGAAATCGTGCG-3′	204
	R: 5′-GTGCCACCAGACAGCACTGTG-3′	

#### Detection of ICAM-1, VCAM-1, IL-1β protein expression in the intima of hyperplastic vessels

The expression of ICAM-1, VCAM-1, IL-1β in the blood vessels with intimal hyperplasia was determined by immunohistochemistry (primary antibody dilution ratio: ICAM-1 1:100, VCAM-1 1:500, IL-1β 1:100, FN 1:100, Col-I 1:100); The operation is carried out according to the instructions of the kit. Under the light microscope, it can be seen that brown-yellow spot or fibrous staining is concentrated in the cell membrane, cytoplasm or between cells, which is positive expression, and negative is no brown-yellow staining. The slices were photographed with MIAS medical image analysis system, and then analyzed with Image-Pro Plus 6.0: under a 400-fold light microscope, three different fields of view were selected for each slice, and the integrated optical density (IOD) of positive staining per unit area was measured, and then the average was taken for statistical analysis.

#### Detection of plasma inflammatory response-related factors

The plasma levels of IL-1β, TNF-α, and MCP-1 were measured by ELISA to reflect the state of systemic inflammatory response. The specific operation is carried out according to the instructions of the kit.

### HPLC methods

#### Sample preparation

DGBXD sample: 1 g of the extract was dissolved in 30 ml of distilled water, ethanol was added to 80%, precipitated, filtered, the filtrate was evaporated to dryness and the volume was adjusted to 100 ml with 70% methanol. Finally, the extract was filtered through a 0.45-μm membrane.

Ferulic acid reference sample: 1.05 mg of ferulic acid was adjusted to 5 ml with 70% methanol, and then 3 ml was accurately measured, and the volume was adjusted to 50 ml with 70% methanol, and the concentration was 0.0126 mg/ml. Finally, it was filtered with a 0.45-μm filter membrane.

#### HPLC condition

**Figure 2:**
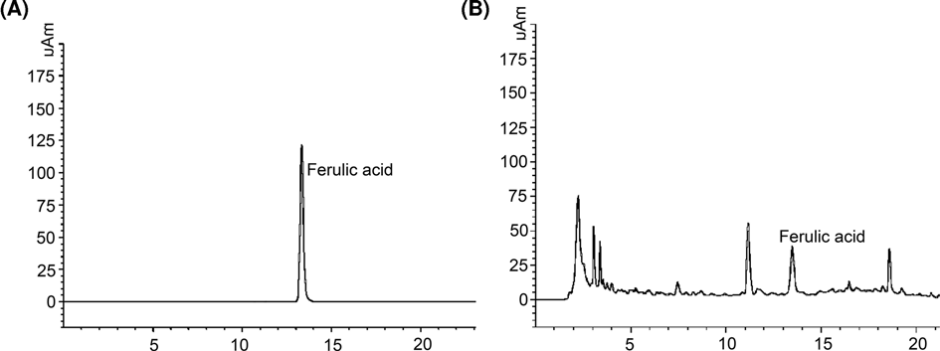
The results of HPLC (**A**) Ferulic acid reference sample; (**B**) DGBXD sample.

Column: Agilent ZORBAX Eclipse XDB-C18 column (250 mm × 4.6 mm, 5 μm); mobile phase A: 0.2% formic acid solution, mobile phase B: 0.2% formic acid acetonitrile solution, flow rate: 10 ml/min; column temperature: 30°C; injection volume: 10 μl. The chromatogram is shown in Figure 2. After determination, the content of ferulic acid in DGBXD is 0.5900 mg/g.

### Statistical analysis

SPSS 17.0 statistical software was used for analysis, and the experimental data were expressed as mean ± SD. One-way ANOVA was used to compare the means among multiple groups. For pairwise comparisons between two groups, if the variances are uniform, the LSD test is used, and if the variances are not uniform, Dunnett’s T3 test is used. *P*<0.05 was considered statistically significant.

## Results and discussion

### The known targets and potential targets of DGBXD and AS genes

**Figure 3:**
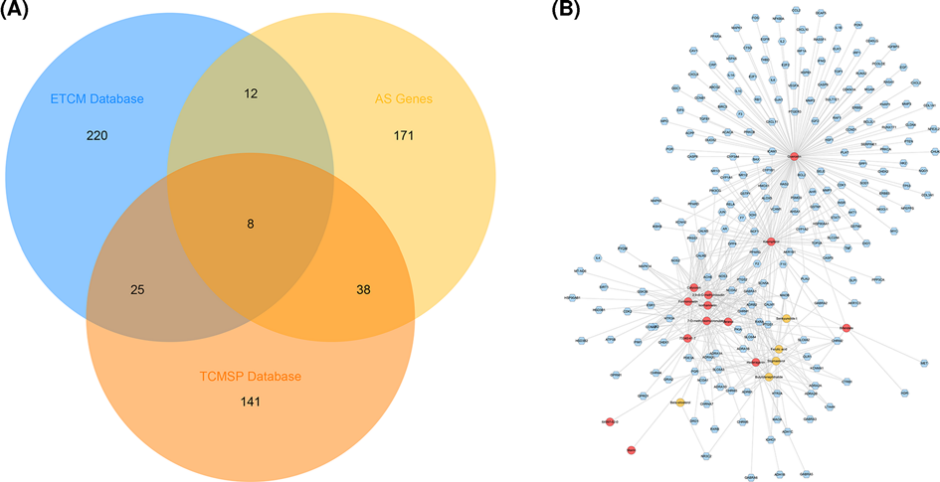
The known targets and potential targets of DGBXD and AS genes (**A**) Venn diagram of known targets and potential targets of DGBXD and AS genes. (**B**) Compound-known target network. Pink hexagon stands for known targets; red and orange circles stand for *Hedysarum Multijugum Maxim.* and *Angelicae Sinensis Radix* components, respectively.

A total of 212 known targets, 265 potential targets, and 229 AS genes were obtained. There is overlap between the target of each target set (Figure 3A). The compounds and known targets were input into Cytoscape to construct compound-known target network, which consists of 18 compound nodes, 212 known target nodes, and 537 edges (Figure 3B). In this network, some targets can be regulated by a lot of compounds (for example, PTGS1 can be regulated by Quercetin, Kaempferol, 7-*o*-methylisomucronulatol, Formononetin, Isorhamnetin, Stigmasterol, 3,9-di-*o*-methylnissolin, 73340-41-7, Butylidenephthalide, Calycosin, Hederagenin, Ferulic acid, Jaranol, Senkyunolide I, Bifendate), while other targets were regulated by only one compound (for example, CASP9 is regulated by Quercetin).

### DGBXD known target-AS PPI network analysis

#### DGBXD known target-AS PPI network construction

**Figure 4:**
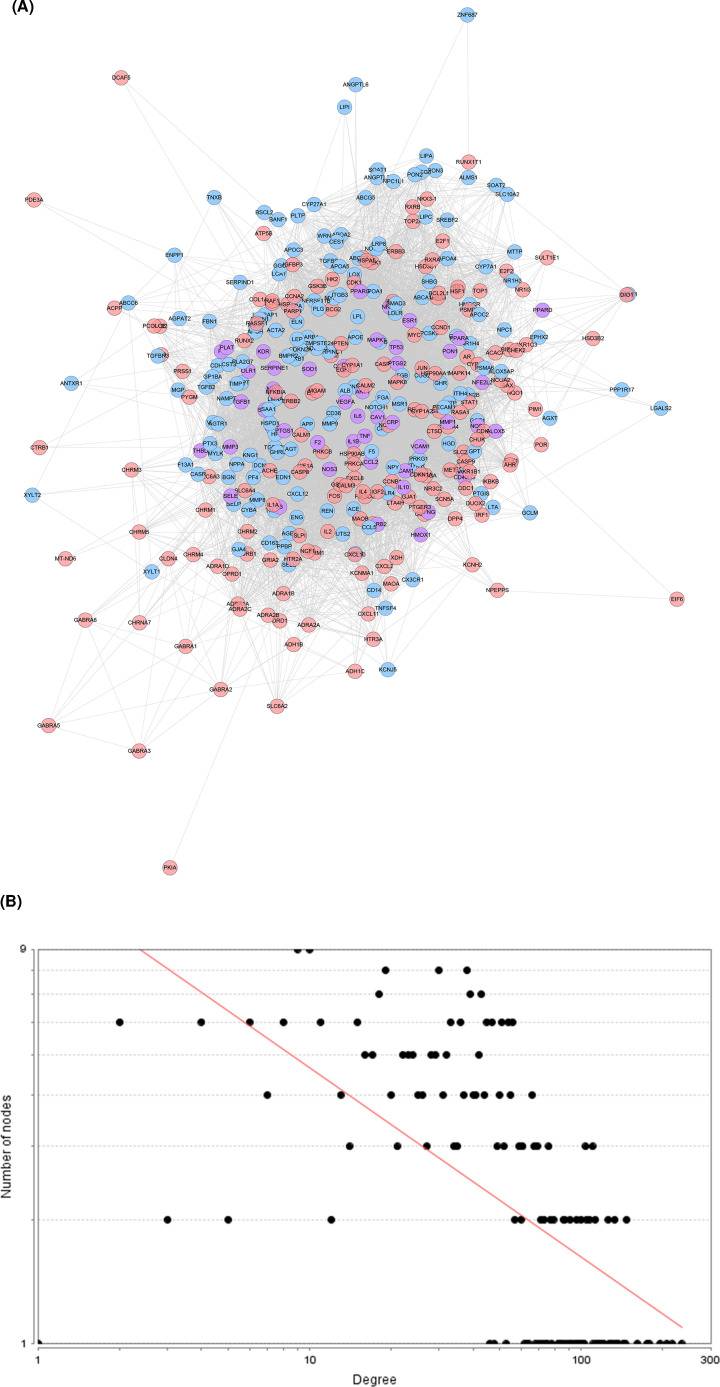
DGBXD known target-AS PPI network (**A**) PPI network. (**B**) Node degree distribution of DGBXD Known Target-AS PPI Network. Pink circles stand for DGBXD known target, blue circles stand for AS genes, purple circles stand for DGBXD-AS target.

The DGBXD Known Target-AS PPI network was composed of 165 DGBXD known target nodes, 162 AS gene nodes, 46 DGBXD known-AS targets and 9094 edges (Figure 4A). The targets are arranged in descending order according to their degree, the top 20 can be divided into three categories: (1) DGBXD known target: CASP3 (147 edges), EGF (145 edges), CXCL8 (144 edges), EGFR (143 edges), MAPK8 (135 edges), JUN (133 edges); (2) AS genes: INS (234 edges), ALB (217 edges), MMP9 (147 edges), APOE (134 edges), APP (133 edges), TLR4 (129 edges); (3) DGBXD known-AS target: IL6 (207 edges), AKT1 (195 edges), TNF (178 edges), VEGFA (175 edges), TP53 (161 edges), CCL2 (137 edges), MAPK1 (136 edges), IL1B (131 edges). The topological property of this network was assessed by network analyzer tool, and the result demonstrates that DGBXD Known Target-AS PPI network meets the power-law distribution (R^2^ = 0.377, y = 13.382x^−0.459^) (Figure 4B).

#### Biological processes of DGBXD known target-AS PPI network

**Figure 5:**
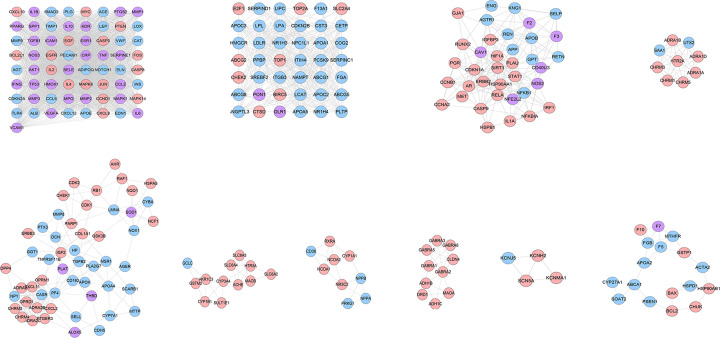
Clusters of DGBXD known target-AS PPI network Pink circles stand for DGBXD known target, blue circles stand for AS genes, purple circles stand for DGBXD-AS target

The DGBXD Known Target-AS PPI network was analyzed by MCODE and ten clusters were obtained (Figure 5 and Table 3). The targets and genes of each cluster were input into DAVID to perform GO enrichment analysis so as to obtain the biological processes of each cluster.

**Table 3:** Clusters of DGBXD known target-AS PPI network

Cluster	Score	Nodes	Edges	Targets and genes
1	54.344	65	1739	PTEN, IL1B, AGT, MPO, CXCL10, HMOX1, SPP1, PTGS2, SMAD3, MMP1, MAPK14, TIMP1, TGFB1, APOE, JUN, IL4, PPARG, CCL2, CRP, SELE, PLG, ESR1, TP53, NOS3, AKT1, IL6, INS, CASP3, TNF, MAPK8, ADIPOQ, ACE, KDR, VCAM1, VEGFA, SERPINE1, MMP9, ICAM1, NOTCH1, TLR4, EDN1, IFNG, CDKN2A, ALB, EGFR, CCND1, MAPK1, BCL2L1, FOS, LEP, EGF, CXCL12, ELN, VWF, CASP8, PECAM1, LOX, MMP2, MYC, CXCL8, MMP3, CCL5, CAT, IL10, IL2
2	13.707	42	281	HMGCR, APOC2, ABCG2, APOC3, PON1, CHEK2, CST3, E2F1, SREBF2, NAMPT, APOA5, CTSD, NR1H4, SLC2A4, PPBP, ITIH4, ITGB3, NR1H3, PLTP, TOP2A, CDKN2B, ABCG1, NPC1L1, COG2, TOP1, LDLR, SERPIND1, APOA1, CETP, LPL, FGA, ABCG8, LIPC, SERPINC1, ABCG5, OLR1, F13A1, BIRC5, PCSK9, LCAT, ANGPTL3, LPA
3	10.111	37	182	AGTR1, RELA, IL1A, HSP90AA1, PGR, STAT1, AR, RUNX2, CAV1, IGFBP3, GPT, IRF1, NOS2, NFE2L2, SELP, NFKB1, CCNA2, CDKN1A, MET, CASP9, NFKBIA, CD40LG, APOB, F2, PLAU, KNG1, HIF1A, ENG, SIRT1, ERBB2, APP, GJA1, RETN, HSPB1, REN, F3, CCNB1
4	9	9	36	HTR2A, ADRA1A, ADRA1B, ADRA1D, UTS2, CHRM3, CHRM1, SAA1, CHRM5
5	6.692	53	174	NQO1, PARP1, OPRM1, CXCL11, CXCL2, CHRM4, OPRD1, CHRM2, IGF2, APOA4, ERBB3, DPP4, CYBA, GSK3B, CDK2, CHEK1, NPY, ADRA2A, HP, ADRA2B, APOH, TGFB2, CDH5, LMNA, PF4, CYP7A1, GGT1, NOX1, CD163, NCF1, PTX3, MSR1, CDK1, SOD1, AHR, THBD, PLAT, MMP8, SCARB1, CASR, AGER, PLA2G7, ALOX5, RB1, RAF1, MTTP, HSPA5, DCN, SELL, PTGER3, TNFRSF11B, ADRA2C, COL1A1
6	5.111	10	23	GABRA1, GABRA2, GABRA3, MAOA, GABRA6, CLDN4, DRD1, GABRA5, ADH1B, ADH1C
7	4.364	12	24	GCLC, ACHE, SLC6A3, SLC6A4, SLC6A2, MAOB, GSTM2, SULT1E1, AKR1C3, CYP3A4, HTR3A, CYP1B1
8	4.25	9	17	RXRA, NCOA1, NR3C2, PRKG1, NPPA, NCOA2, NPPB, CD36, CYP1A1
9	3.333	4	5	KCNJ5, KCNH2, SCN5A, KCNMA1
10	3.25	17	26	CYP27A1, FGB, ACTA2, BCL2, BAX, PSEN1, F5, CHUK, F10, ABCA1, HSP90AB1, SOAT2, GSTP1, HSPD1, F7, MTHFR, APOA2

**Figure 6:**
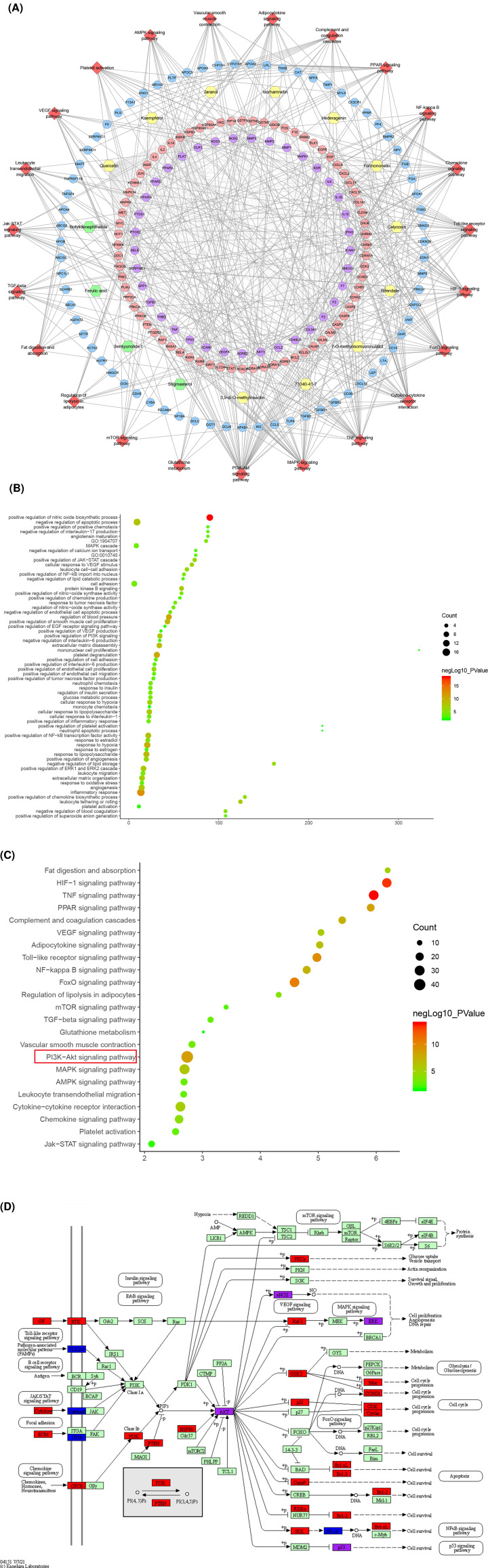
Enrichment analysis results (**A**) Component-known target-pathway network; red diamonds stand for signaling pathways. Pink circles stand for DGBXD known target, blue circles stand for AS genes, purple circles stand for DGBXD-AS target. Yellow and green hexagons stand for *Hedysarum Multijugum Maxim.* and *Angelicae Sinensis Radix* components, respectively. (**B**) Bubble chart of biological processes in cluster 1. (**C**) Bubble chart of signaling pathway. X-axis stand for fold enrichment. (**D**) PI3K-AKT signaling pathway modified from has04151.

Cluster 1 is related to inflammation, platelet activation, endothelial cell apoptosis, oxidative stress, lipid metabolism, vascular smooth muscle proliferation, angiogenesis, NFKB signaling pathway, leukocyte migration and rolling. Cluster 2 is related to lipid metabolism such as cholesterol and triglycerides, foam cell differentiation, and platelet degranulation. Cluster 3 is related to angiogenesis, endothelial cell proliferation, active oxygen metabolism, foam cell differentiation, oxidative stress, hypoxia, and cholesterol metabolism. Cluster 4 is related to vascular smooth muscle contraction. Cluster 5 is related to inflammatory chemotaxis, blood coagulation, oxidative stress, and cholesterol metabolism. Cluster 7 is related to steroid metabolism and redox. Cluster 10 is related to endoplasmic reticulum stress, cholesterol efflux, coagulation, and hypoxia. Clusters 6, 8, and 9 failed to return any AS-related biological processes (Supplementary Table S4). The *P*-value, fold enrichment, and count of biological processes in cluster 1 were shown in Figure 6B as an example.

#### Pathway of DGBXD known target-AS PPI network

The pathway enrichment analysis showed that DGBXD can regulate a lot of AS-related signaling pathways, such as TNF signaling pathway, HIF-1 signaling pathway, FoxO signaling pathway, Toll-like receptor signaling pathway, PI3K-Akt signaling pathway, PPAR signaling pathway, nuclear factor κB (NF-κB) signaling pathway, Complement and coagulation cascades, Adipocytokine signaling pathway, mitogen-activated protein kinase (MAPK) signaling pathway (Figure 6A and Supplementary Table S5). The *P*-value, fold enrichment, and count of each signaling pathways were shown in Figure 6C. The PI3K-Akt signaling pathway was shown in Figure 6D. The DGBXD potential targets were marked in red; the AS genes were marked in blue; the DGBXD-AS targets were marked in purple.

### DGBXD-AS PPI network analysis

#### DGBXD-AS PPI network construction

**Figure 7:**
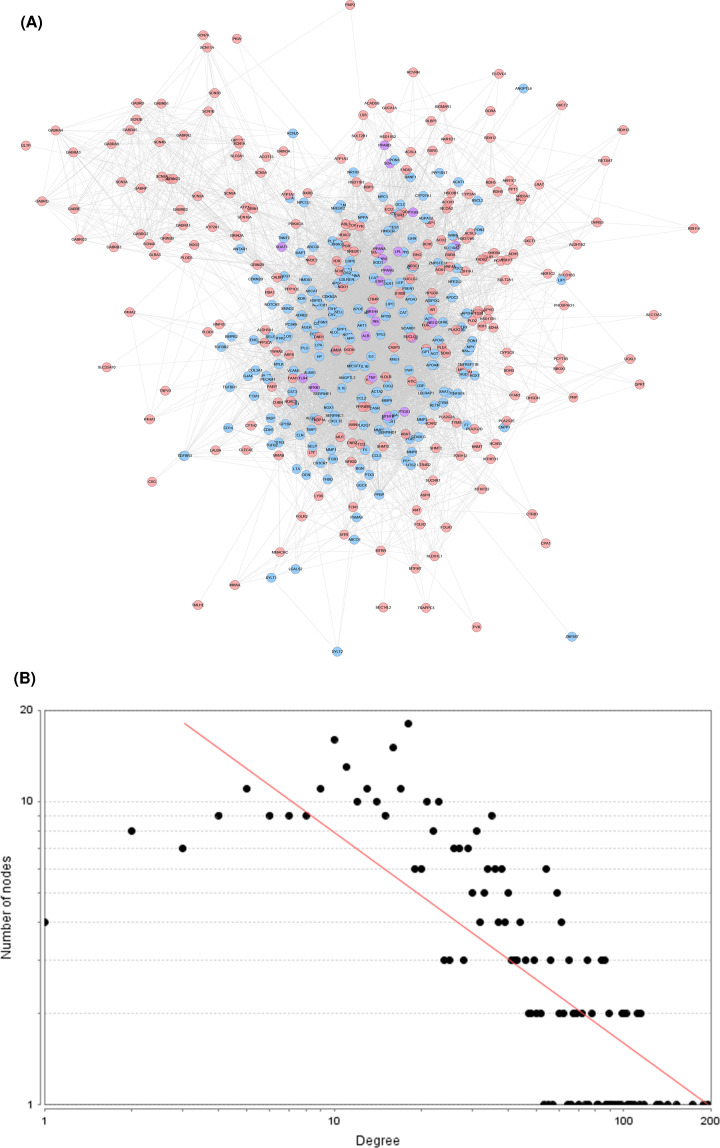
DGBXD-AS PPI network (**A**) PPI network. (**B**) Node degree distribution of DGBXD-AS PPI network. Pink circles stand for DGBXD target, blue circles stand for AS genes, purple circles stand for DGBXD-AS target.

The DGBXD-AS PPI network was composed of 225 DGBXD target nodes, 188 AS gene nodes, 20 DGBXD-AS targets and 7081 edges (Figure 7A). The targets are arranged in descending order according to their degree, the top 21 can be divided into three categories: (1) DGBXD target: CASP3 (102 edges); (2) AS genes: IL6 (173 edges), AKT1 (153 edges), VEGFA (134 edges), APOE (130 edges), APP (118 edges), MAPK1 (115 edges), CCL2 (115 edges), TP53 (114 edges), IL1B (112 edges), APOB (112 edges), MMP9 (109 edges), IL10 (105 edges), CRP (103 edges), NOS3 (102 edges), LEP (100 edges), SERPINE1 (100 edges); (3) DGBXD-AS target: INS (196 edges), ALB (189 edges), TNF (142 edges), TLR4 (106 edges). The topological property of this network was assessed by network analyzer tool, and the result demonstrates that DGBXD-AS PPI network meets the power-law distribution (R^2^ = 0.611, y = 38.943x^−0.694^) (Figure 7B).

#### Biological processes of DGBXD-AS PPI network

**Figure 8:**
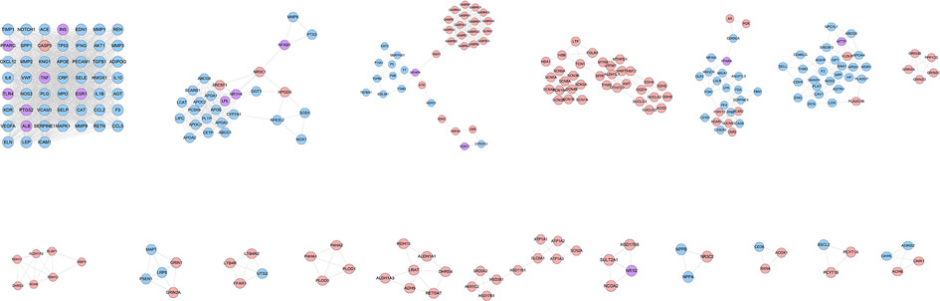
Clusters of DGBXD-AS PPI network Pink circles stand for DGBXD target, blue circles stand for AS genes, purple circles stand for DGBXD-AS target.

The DGBXD-AS PPI network was analyzed by MCODE and 18 clusters were obtained (Figure 8 and Table 4). The targets and genes of top ten clusters were input into DAVID to perform GO enrichment analysis so as to obtain the biological processes of each cluster.

**Table 4:** Clusters of DGBXD-AS PPI network

Cluster	Score	Nodes	Edges	Targets and genes
1	45.373	52	1157	TP53, AKT1, CCL2, CRP, SELE, KDR, NOS3, NOTCH1, IL6, ADIPOQ, ACE, IFNG, VCAM1, VEGFA, SERPINE1, MMP9, ICAM1, MAPK1, EDN1, CXCL12, SELP, LEP, ELN, VWF, CASP3, PPARG, PECAM1, MMP2, CCL5, RETN, MMP3, CAT, IL10, F3, TNF, IL1B, AGT, MPO, HMOX1, SPP1, PTGS2, MMP1, TIMP1, TGFB1, INS, ESR1, TLR4, KNG1, ALB, APOE, PLG, REN
2	12.231	27	159	CETP, ABCG8, LIPC, PTX3, CYP7A1, PCSK9, LCAT, APOA2, SOD1, APOC2, APOC3, LPL, SREBF1, MMP8, NFE2L2, APOA5, SCARB1, PLTP, NR1H4, ABCG1, NR3C1, HPGDS, NFKB1, GGT1, NOX1, APOB, APOA1
3	9.657	36	169	SOAT2, GABRQ, FGB, ATIC, F5, CST3, GABRB1, CYP27A1, GABRA1, GABRB2, GABRA2, GABRB3, GABRA3, MTHFR, GABRA4, GABRA5, HNF4A, GABRD, GABRE, GABRA6, SDHC, TGFBR1, ITGB3, SERPIND1, TGFB2, GLRA3, GABRG1, NQO1, HSPD1, GABRG2, F13A1, COL3A1, GABRG3, F7, VDR, GABRP
4	8.242	34	136	SCN1A, FOLR3, SCN1B, MTHFD1, MTRR, HBB, MTHFD2, HBA1, OGDH, ACO2, SCN2B, SCN3A, TCN1, SCN3B, SCN4A, SCN10A, MTFMT, SCN4B, SUCLG1, ALDH1L1, SUCLG2, SCN5A, SDHA, SDHB, AMT, SDHD, LTF, TYMS, MTR, SUCLA2, SHMT1, SCN8A, SHMT2, SCN9A
5	6.833	25	82	ABCA1, ANGPTL3, CD163, OLR1, CX3CR1, LPA, HMGCR, HCAR2, PON1, P2RY12, HCAR3, CDKN2A, SUCNR1, CASR, PPBP, PPARA, NR1H3, FBN1, AR, FGA, PF4, SERPINC1, PGR, LDLR, CNR2
6	5.926	28	80	ABCG5, AGTR1, MSR1, SAA1, MTTP, SREBF2, GPT, CAV1, SMAD3, NOS2, APOA4, THBD, PLAT, HP, AGER, NPC1L1, PLA2G7, CD40LG, APOH, DCN, APP, CDH5, SELL, F2, LOX, VLDLR, ENG, PLA2G1B
7	5	5	10	GRIN2C, PPP1CC, GRIN3B, GRIN2D, GRIN3A
8	4.667	7	14	RLBP1, RBP3, RDH11, DHRS3, RDH12, RDH8, ALDH1A2
9	4.5	5	9	PSEN1, GRIN2A, LRP8, GRIN1, MAPT
10	4	4	6	FFAR1, LTB4R2, LTB4R, UTS2
11	4	4	6	P4HA1, PLOD3, PLOD1, P4HA2
12	4	7	12	ADH5, RETSAT, LRAT, ALDH1A1, RDH13, DHRS4, ALDH1A3
13	3.556	10	16	HSD11B1, HSD3B1, SRD5A2, AKR1C2, HSD17B1, SLC8A1, ATP1A1, ATP1A2, SCN2A, ATP1A3
14	3.333	4	5	HSD17B6, NR1I2, NCOA2, SULT2A1
15	3	3	3	NR3C2, NPPB, NPPA
16	3	3	3	CD36, ACOX1, RXRA
17	3	3	3	PCYT1A, PCYT1B, BSCL2
18	2.667	4	4	ADRB2, CNR1, GHRL, ACHE

**Figure 9:**
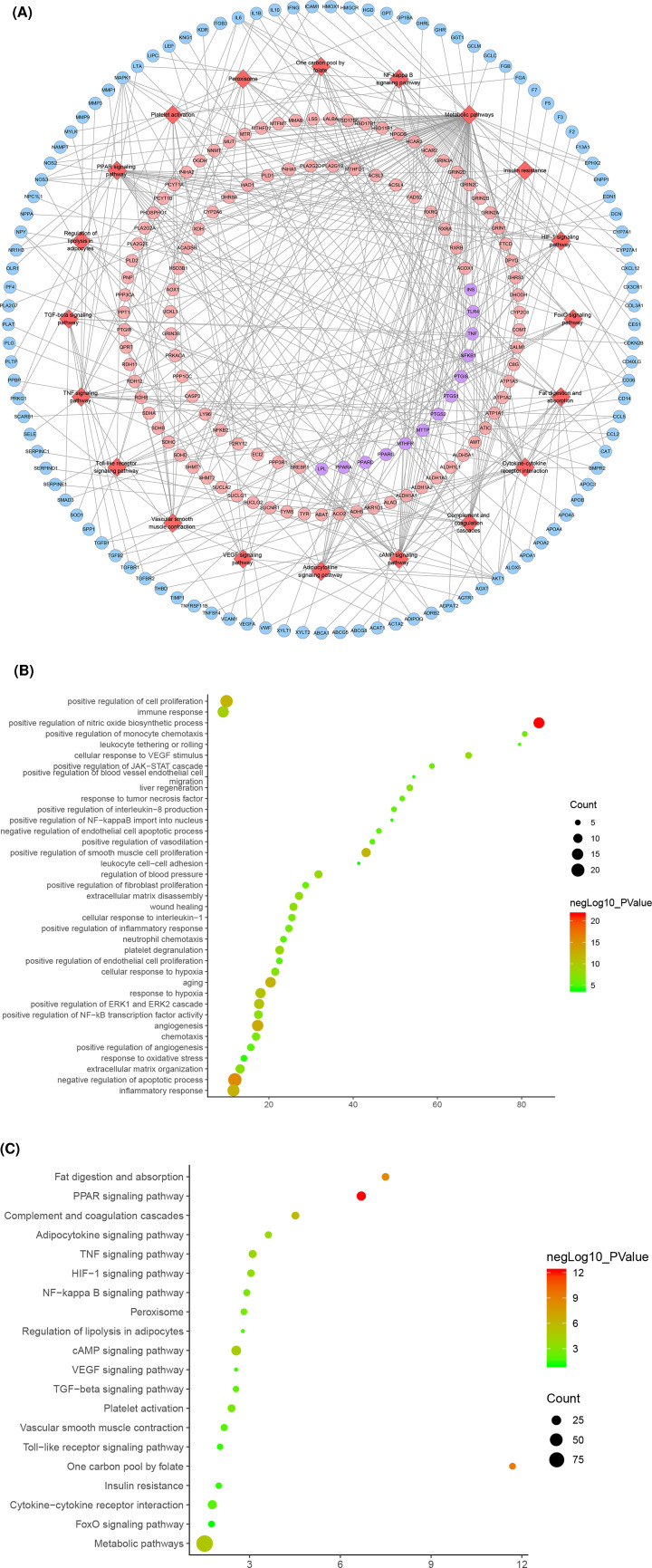
Enrichment analysis results (**A**) Pathways of DGBXD-AS PPI network; red diamonds stand for signaling pathways, pink circles stand for DGBXD target, blue circles stand for AS genes, purple circles stand for DGBXD-AS target. (**B**) Bubble chart of biological processes in cluster 1. (**C**) Bubble chart of signaling pathway. X-axis stands for fold enrichment.

Cluster 1 is related to apoptosis, inflammatory chemokines, and their mediated severe inflammatory response, smooth muscle proliferation, hypoxia, endothelial cell proliferation and apoptosis, vasodilation, and oxidative stress. Cluster 2 is related to cholesterol and other lipid anabolism and inflammation. Cluster 3 is related to coagulation reaction and hypoxia reaction. Cluster 5 is related to cholesterol metabolism, macrophage foam cell transformation, inflammatory chemotaxis, and blood coagulation. Cluster 6 is related to hypoxia, cholesterol metabolism, and blood coagulation. Cluster 7 is related to calcium ion transport across membranes. Cluster 9 is related to calcium ions. Clusters 4, 8, and 10 failed to return any AS-related biological processes (Supplementary Table S6). The *P*-value, fold enrichment, and count of biological processes in Cluster 1 were shown in Figure 9B as an example.

#### Pathway of DGBXD-AS PPI network

The pathway enrichment analysis showed that DGBXD can regulate a lot of AS-related signaling pathways, such as PPAR signaling pathway, One carbon pool by folate, Fat digestion and absorption, Complement and coagulation cascades, Metabolic pathways, cAMP signaling pathway, TNF signaling pathway, Adipocytokine signaling pathway, HIF-1 signaling pathway, NF-κB signaling pathway (Figure 9A and Supplementary Table S7). The *P*-value, fold enrichment, and count of each signaling pathways were shown in Figure 9C.

The results of network pharmacology suggest that DGBXD may have anti-AS effects. Astragaloside IV is the main medicinal substance of *Hedysarum Multijugum Maxim.*, which has cardiovascular protective effects such as strengthening the heart, protecting myocardial cells, protecting vascular endothelial cells, inhibiting the proliferation of vascular smooth muscle cells (VSMCs), and regulating blood pressure; its mechanism is related to anti-oxidation, scavenging free radicals, anti-inflammatory, anti-apoptosis, etc [31,32]. Current studies also show that stigmasterol can regulate fatty acid synthesis and cholesterol metabolism, lipid metabolism, inhibit inflammation, and thus play a cardiovascular protective effect [33]. Current research shows that Butylidenephthalide has its anti-platelet activity [34], as well as anti-tumor [35] and anti-inflammatory properties [36]. 3,9-di-*o*-methylnissolin belongs to isoflavones. Current research shows that isoflavones have good antioxidant and anti-inflammatory functions [37]. Studies have also shown that Calycosin can inhibit inflammation through NF-KB signaling pathway and MAPK signaling pathway [38,39]. Ferulic acid has many physiological functions (anti-inflammatory, antioxidant, antidiabetic effect and free radical scavenger, scavenging lipids) [40–42]. The current research progress shows its anti-fatty liver effect. Formononetin has anti-inflammatory properties [43]. In the process of protecting endothelial damage, Formononetin improves the endothelial dysfunction induced by high glucose by inhibiting the JAK/STAT signaling pathway [44]. In terms of oxidative stress, Hederagenin prevents AS by inhibiting the Nrf2-ARE antioxidant pathway [45,46]. Current research shows that Isorhamnetin has a wide range of pharmacological activities, such as protecting cardiovascular and cerebrovascular, anti-tumor, anti-inflammatory, antioxidant, organ protection, and preventing obesity. The mechanism involves PI3K/AKT/PKB pathway, NF-κB pathway, MAPK pathway and other signal pathways, as well as the expression of related cytokines and kinases [47–49]. A large number of preclinical studies have shown that Kaempferol has antioxidant, anti-inflammatory, antimicrobial, antitumor, cardioprotective, neuroprotective, antidiabetic, anti-osteoporotic, and estrogen/anti-estrogen effects [50,51]. As a flavonoid, quercetin has been shown to have significant heart-related benefits, such as inhibiting LDL oxidation, non-endothelial-dependent vasodilation, reducing adhesion molecules and other inflammatory markers, and protecting endothelial function [52,53]. Senkyunolide I is one of the biologically active ingredients. Current studies have shown that it has anti-inflammatory, antioxidative damage [54], anti-platelet, anti-coagulation [55], and alleviates cerebral ischemia–reperfusion injury [56]. Therefore, it is speculated that the above active ingredients may be the key ingredients of DGBXD anti-AS.

The results of GO and pathway enrichment analysis showed that DGBXD can mainly regulate inflammatory chemokines and their mediated signal transduction, blood coagulation, smooth muscle proliferation, endothelial cell proliferation, angiogenesis, leukocyte adhesion, migration and activation, oxidative stress, and other biological modules. The signaling pathways that DGBXD can regulate mainly include PI3K/Akt signal pathway, TNF signal pathway, NF-κB signal pathway, HIF-1 signal pathway, FoxO signal pathway/PPAR signal pathway, etc. Current research shows that AS is a chronic inflammatory disease of the blood vessel wall, and the inflammatory response plays an important role in different stages of AS [57,58]. Cytokines, which are important mediators of inflammation, can be secreted by a variety of activated cells in AS. At the same time, these cytokines can activate different types of cells and play a key role in AS [59]. Cytokines realize biological activity through their related signal pathways. These pathways include PI3K/AKT signaling pathway, nuclear factor-κB pathway, TGF-β/Smad signaling pathway, JAK/STATA signaling pathway, HIF signaling pathway and Toll signaling pathway, MAPK signaling pathway, etc [60–63]. The above results suggest that DGBXD can act on multiple signal pathways to play an anti-AS effect through a variety of biological processes. There is a complex interaction relationship between these pathways, which reflects the characteristics of multicomponent, multitarget, and multipath cooperative treatment of diseases in TCM. Next, we would further verify the prediction results of network pharmacology through animal experiments.

### Effect of DGBXD on the pathological morphology of rat vascular intima

**Figure 10:**
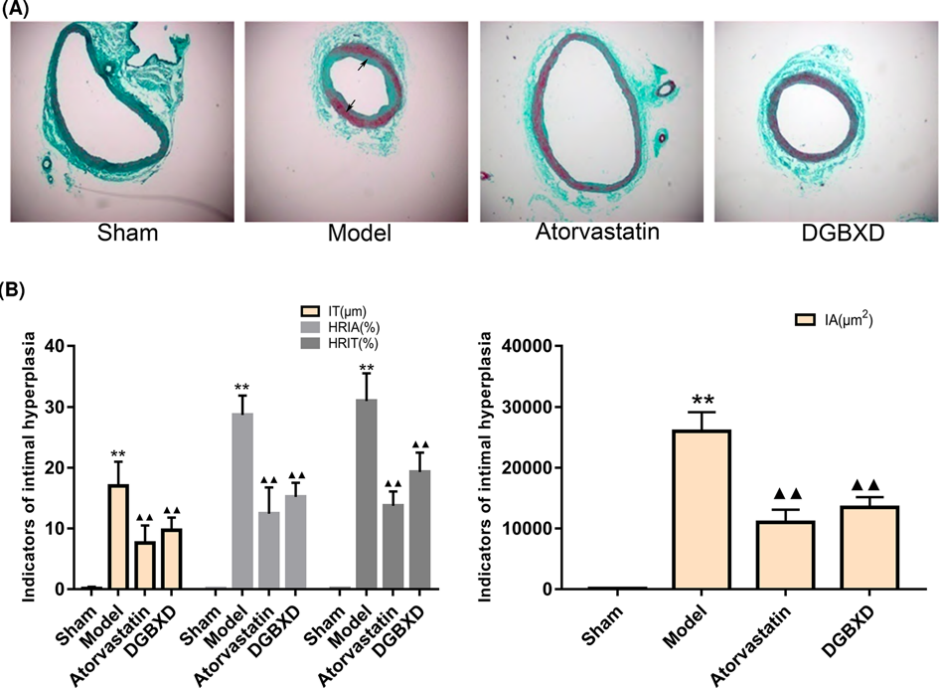
Pathological morphology changes of rat vascular intima Effect of DGBXD on the pathological morphology of rat vascular intima (**A**) pathological morphology; Masson’s staining, ×100. The black arrow points to the hyperplasia. (**B**) Comparison of vascular intimal hyperplasia; compared with sham operation group, **P<0.01; compared with model group, ^▲▲^*P*<0.01.

In the sham operation group, the elastic membrane in the vascular intima was intact, in a single layer, and there was no hyperplasia. In the model group, the angiogenesis intima showed uniform or uneven thickening, a large number of proliferated VSMCs existed, the arrangement was disordered, the lumen showed centripetal or eccentric stenosis, and the intimal hyperplasia was obvious. In the DGBXD group and the atorvastatin group, the vascular intima showed proliferative changes, but the degree of proliferation was less than that of the model group (Figure 10A).

The vascular morphometric analysis showed that compared with the sham operation group, the vascular IA, IT, HRIA, and HRIT of the model group increased significantly (*P*<0.01), indicating that the model was successful. The rat thoracoabdominal aorta showed obvious intimal hyperplasia after balloon injury. Compared with the model group, the vascular tissue IA, IT, HRIA, and HRIT of the DGBXD group and the atorvastatin group were significantly reduced (*P*<0.01) (Figure 10B).

### Expression of ICAM-1, VCAM-1, and IL-1β protein in blood vessels

**Figure 11:**
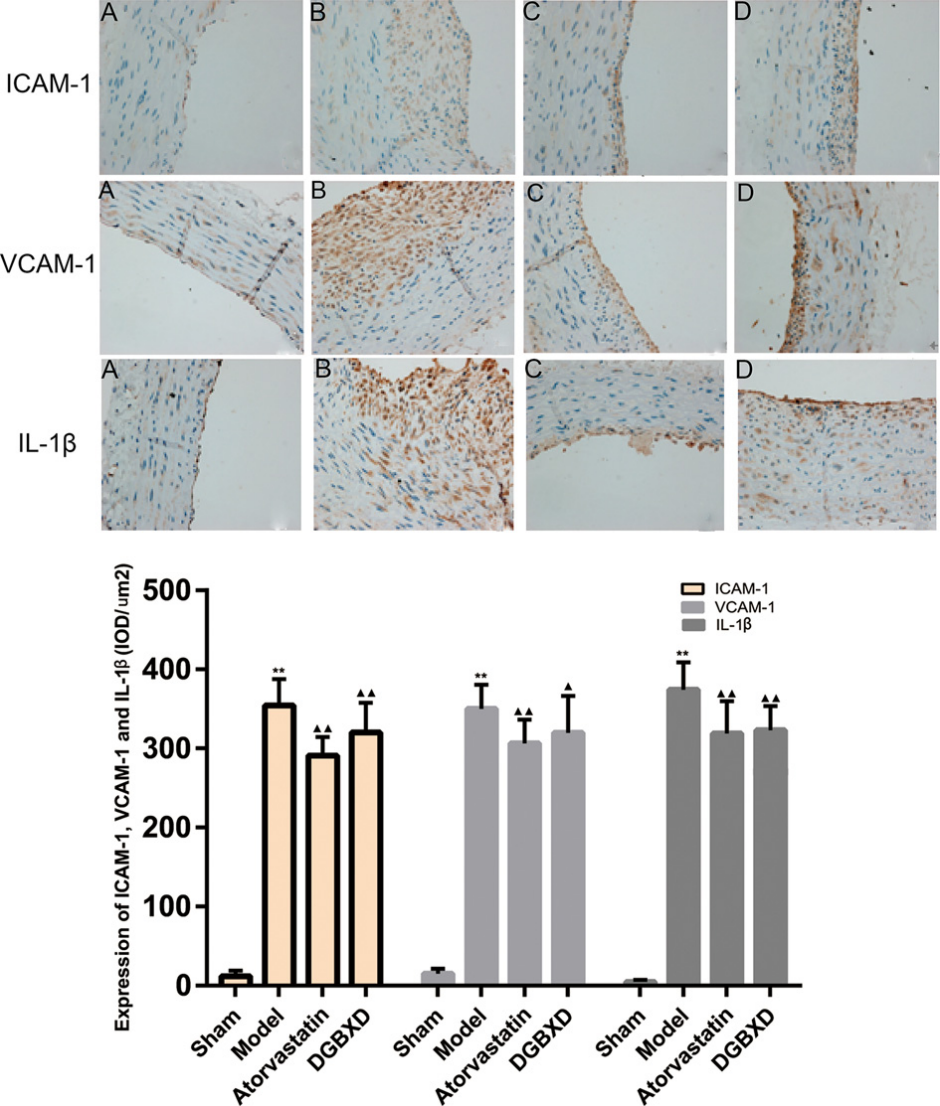
ICAM-1, VCAM-1, and IL-1β Expression in blood vessels Expression of ICAM-1, VCAM-1, and IL-1β in blood vessels (**A**) sham operation group; (**B**) model group; (**C**) atorvastatin group; (**D**) DGBXD group. Immunohistochemistry, ×400. Compared with sham operation group, ** *P*<0.01; compared with model group, ^▲^*P*<0.05, ^▲▲^*P*<0.01

Compared with the sham operation group, the expression of ICAM-1, VCAM-1, and IL-1β in the local blood vessels of the model group was significantly increased (*P*<0.01). Compared with the model group, the vascular ICAM-1, VCAM-1, and IL-1β expression intensity in the atorvastatin group and DGBXD group were significantly lower than those in the model group (*P*<0.01, *P*<0.05). There was no statistical difference of ICAM-1, VCAM-1, and IL-1β between the DGBXD group and the atorvastatin group (*P*>0.05) (Figure 11).

### Expression of ICAM-1, VCAM-1, and IL-1β mRNA in blood vessels

**Figure 12:**
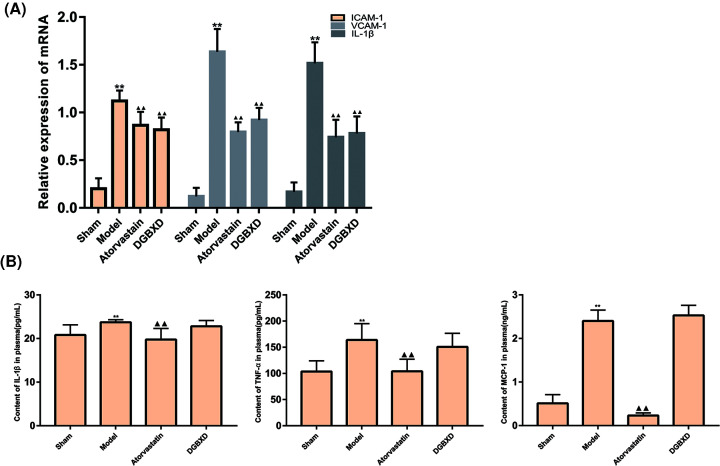
Inflammatory factor expression (**A**) Expression of ICAM-1, VCAM-1, and IL-1β mRNA. (**B**) Plasma inflammatory factors IL-1β, TNF-α, MCP-1 content compared with sham operation group, **P<0.01; compared with model group, ^▲^*P*<0.05, ^▲▲^*P*<0.01.

Compared with the sham operation group, the expression of ICAM-1, VCAM-1, and IL-1β mRNA in the blood vessels of the model group was significantly increased (*P*<0.01). Compared with the model group, the vascular ICAM-1, VCAM-1, and IL-1β mRNA expression intensity in the atorvastatin group and DGBXD group were significantly lower than those in the model group (*P*<0.01, *P*<0.05). There was no statistical difference of ICAM-1, VCAM-1, and IL-1β mRNA between the DGBXD group and the atorvastatin group (*P*>0.05) (Figure 12A).

### Detection of plasma inflammatory factors IL-1β, TNF-α, MCP-1 content

Compared with the sham operation group, the plasma levels of IL1β, TNF-α, and MCP-1 in the model group were significantly increased (*P*<0.01). Compared with the model group, the plasma levels of IL-1β, TNF-α, and MCP-1 in the atorvastatin group were significantly reduced (*P*<0.01). Compared with the atorvastatin group, the plasma levels of IL-1β, TNF-α, and MCP-1 in the DGBXD group were significantly increased (*P*<0.01) (Figure 12B).

### Molecular docking results of DGBXD components and PIK3R1 and AKT1

**Figure 13:**
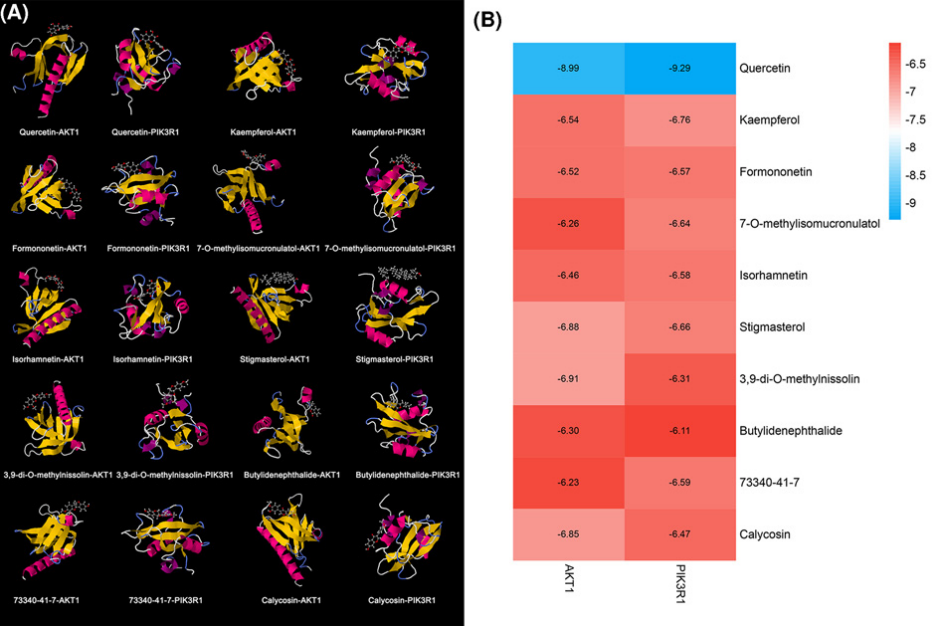
Molecular docking results (**A**) Molecule docking pattern diagram. (**B**) Binding energy [kcal/mol].

The top ten DGBXD components in compound-known target network were selected for molecular docking. The results show that the top ten components may be stably combined with PIK3R1 and AKT1 (Figure 13). This suggests that DGBXD may act on PIK3R1 and Akt through these active components, thereby regulating the PI3K-Akt signaling pathway.

Current research shows that the main biological process of AS is the inflammatory response of the vascular intima. After vascular intima injury, the inflammatory cells are activated and the blood vessels are mechanically expanded, which increases the release of inflammatory factors and chemotactic factors, and exposes the subintimal tissues, induces platelet adhesion and aggregation at the damaged intima, forming a platelet covering layer [64–66]. The activated platelets adhere to circulating white blood cells through platelet receptors, and mediate white blood cells to roll along the damaged endothelial surface. Damaged endothelial cells, VSMCs, and activated inflammatory cells secrete inflammatory chemokines and inflammatory mediators through the adherent platelet–fibrin layer and exudate to the intima, causing an inflammatory reaction in the blood vessel wall [67,68]. Cytokines and inflammatory response mediators can also induce monocytes, lymphocytes, and other inflammatory cells to chemotaxis to the injury site, resulting in the adsorption of a large number of monocytes and leukocytes on the surface of blood vessels, which induces an early inflammatory response [69,70]. In the following days to weeks, macrophages infiltrate the vascular intima and cluster around the scaffold. Platelets, macrophages, and histiocytes gathered at the injured site secrete a large number of chemokines and growth factors to induce VSMCs to migrate from the media into the inner membrane, and the VSMC changes from the contractile type to the synthetic type. VSMCs proliferate in large quantities, synthesizes a large amount of extracellular matrix (ECM) components and deposits on the vascular wall, forming a neointima [71,72]. VSMCs can maintain its activation state in the inflammatory environment of the intima, continuously synthesize cytokines, growth factors, and ECM [73,74]. In addition, under the influence of various factors such as leukocyte, platelet aggregation, and vascular intima injury, it can cause the increase in the expression of various inflammatory response factor ligands such as macrophage surface antigen-1 and L-selectin receptor on the surface of leukocytes [72,73]. Mediated by pro-inflammatory response mediators such as IL-6, IL-1, MCP-1, TNF-α, ICAM-1 and VCAM-1, local inflammatory reactions can develop It is a systemic inflammatory reaction [74,75]. Therefore, when the vascular intima is injured, the inflammatory reaction can also aggravate the vascular intimal hyperplasia.

In the occurrence of AS, local inflammatory reaction is the cause, and vascular intimal hyperplasia is the result, which constitutes a causal relationship. Current studies have found that some natural active ingredients can inhibit the cascade of inflammatory reactions, inhibit inflammatory stress, and counter-inflammatory adverse events. For example, ligustrazine can inhibit endothelial cell inflammation and leukocyte adhesion response induced by oxidized low-density lipoprotein (ox-LDL), and inhibit the activation of MAPK and NF-κB signaling pathways, which can inhibit the inflammatory response in the initial stage of inflammatory response [76–78]. Quercetin [79], emodin [80], triptolide [81], icariin [82], and total ginsenosides [83] also have similar effects. Lin et al. [84] showed that DGBXD may improve inflammation in AS mice by affecting the activity of NF-kB. Ma et al. [85] and Huang et al. [86] used immunoblotting to detect the effect of DGBXD on the expression of p38 MAPK in RAW264.7 cells activated by ox-LDL. They speculated that the mechanism of DGBXD’s prevention and treatment of AS and related diseases may be to down-regulate the activity of p38 MAPK, thereby blocking the cascade of this pathway to reduce the AS inflammatory response. This prevents ox-LDL from inducing monocytes to accumulate to the vascular endothelium, which not only blocks the early stage of development of AS lesions, but also alleviates the progression of the disease.

The present study found that after balloon catheter injury, the expression of inflammatory response factors ICAM-1, VCAM-1, and IL-1β in the locally hyperplastic intima was significantly increased. It shows that the vascular intima is injured and produces a local inflammatory response of the blood vessel, which can promote the proliferation and migration of VSMC and cause intimal hyperplasia. Atorvastatin and DGBXD can inhibit the expression of ICAM-1, VCAM-1, and IL-1β in the vascular intima, indicating that atorvastatin and DGBXD can reduce the local inflammatory response of blood vessels by inhibiting the expression of local inflammatory response factors in blood vessels. Studies have also found that the local inflammatory response after vascular injury can induce systemic chronic inflammatory response, which in turn promotes the migration and proliferation of VSMCs and causes intimal hyperplasia [87,88]. In addition, atorvastatin can inhibit the increase in plasma IL-1β, TNF-α, and MCP-1 levels, indicating that atorvastatin can inhibit the systemic chronic inflammatory response after vascular intimal injury, while DGBXD does not seem to inhibit systemic inflammation.

## Conclusion

The mechanism of DGBXD in the treatment of AS may be related to the improvement of ECM deposition in the blood vessel wall and the anti-vascular local inflammatory response. This may provide a reference for the study of the mechanism of DGBXD.

## Supplementary Material

Supplementary Tables S1-S7

## Data Availability

All datasets for the present study are included in the manuscript and the supplementary files.

## Abbreviations

AS: atherosclerosis
DAB: Diaminobenzidine
DGBXD: Danggui Buxue Decoction
ECM: extracellular matrix
GO: Gene Ontology
HIF: hypoxia inducible factor-1
HPLC: high performance liquid chromatography
HRIA: hyperplasia ratio of intimal area
HRIT: hyperplasia ratio of intimal thickness
IA: intimal area
ICAM-1: intercellular cell adhesion molecule-1
IL: interleukin
IT: intimal thickness
MAPK: mitogen-activated protein kinase
MCP-1: monocyte chemotactic protein-1
NF-κB: nuclear factor κB
OB: oral bioavailability
ox-LDL: oxidized low-density lipoprotein
TCM: traditional Chinese medicine
TNF-α: tumor necrosis factor-α
VCAM-1: vascular cell adhesion molecule-1
VSMC: vascular smooth muscle cell
